# Barriers to post-abortion care service provision: A cross-sectional analysis in Burkina Faso, Kenya and Nigeria

**DOI:** 10.1371/journal.pgph.0001862

**Published:** 2024-03-07

**Authors:** Winstoun Muga, Kenneth Juma, Sherine Athero, Grace Kimemia, Martin Bangha, Ramatou Ouedraogo

**Affiliations:** African Population Health and Research Center, Nairobi, Kenya; Dalhousie University, CANADA

## Abstract

Despite several political commitments to ensure the availability of and access to post-abortion care services, women in sub-Saharan Africa still struggle to access quality post-abortion care, and with devastating social and economic consequences. Expanding access to post-abortion care while eliminating barriers to utilization could significantly reduce abortions-related morbidity and mortality. We describe the barriers to providing and utilizing post-abortion care across health facilities in Burkina Faso, Kenya, and Nigeria. This paper draws on three data sources: health facility assessment data, patient-exit interview data, and qualitative interviews conducted with healthcare providers and policymakers. All data were based on a cross-sectional survey of a nationally representative sample of health facilities conducted between November 2018 and February 2019. Data on post-abortion care service indicators were collected, including staffing levels and staff training, availability of post-abortion care supplies, equipment and commodities. Patient-exit interviews focused on patients treated for post-abortion complications. In-depth interviews were conducted with healthcare providers within a sample of the study health facilities and national or local decision-makers in sexual and reproductive health. Few primary-level facilities in Burkina Faso (15%), Kenya (46%), and Nigeria (20%) had staff trained on post-abortion care. Only 16.6% of facilities in Kenya had functional operating theaters or MVA rooms, Burkina Faso (20.3%) and Nigeria (50.7%). Primary facilities refer post-abortion care cases to higher-level facilities despite needing to be more adequately equipped to facilitate these referrals. Several challenges that impede the provision of quality and comprehensive post-abortion care across the three countries. The absence of post-abortion care training, equipment, and inadequate referral capacity was among the critical reasons for the lack of services. There is a need to strengthen post-abortion care services across all levels of the health system, but especially at lower-level facilities where most patients seek care first.

## Introduction

In contexts where abortion laws are largely restrictive and considerable proportions of pregnancies are unintended, the majority of women in need of abortion services resort to unsafe methods and procedures [1–4]. Consequently, countries worldwide where abortions are tightly regulated and criminalized are associated with higher abortion-related morbidity and mortality rates [5]. Post-abortion care (PAC) is a lifesaving intervention that focuses on the management of unsafe abortion complications and the provision of post-abortion contraceptives and other reproductive health services [3]. A review by Izugbara et al. (2019) illustrated a complex set of socio-economic (high cost of care), infrastructural (low capacity of health facilities to deliver PAC services), cultural and political factors (including stigma, negative patient-provider interactions that impede access and utilization of PAC services [6]. As a consequence, unsafe abortion is responsible for between 4–13% of all maternal deaths in Africa and disabilities or complications that require various degrees of care [7].

Despite long-standing governmental commitments to ensure availability and access to quality PAC services [8,9], women still face insurmountable challenges to access PAC services in much of sub-Saharan Africa (SSA) [10]. For instance, in Burkina Faso, it is estimated that 22 induced abortions occurred for every 1000 women aged between 15–49 in 2020 [11]. In Burkina Faso still, an estimated 105,000 unsafe abortions occurred in 2012 alone and over 40% of these women experienced complications needing treatment in hospitals, the majority of them did not receive the appropriate medical care they needed [12,13]. In Kenya, about 500,000 induced abortions occurred in 2012, and 75% of these were unsafe, with a considerable proportion resulting in severe complications and, ultimately, deaths [14,15]. About 1.25 million induced abortions occurred in Nigeria in 2012, and of these, 285,000 had serious complications but did not receive the treatment they needed [12,16].

At the health system level, several studies have cited factors such as scarcity of adequately trained health providers, policies that restrict mid-level health providers from offering PAC services or prescribing specific contraception methods, stock-outs of PAC supplies and commodities, and unfavorable attitudes exhibited by providers towards patients, as critical impediments to the delivery of PAC services [17,18].

This is sufficient evidence that expanding high-quality PAC services to primary-level facilities and eliminating barriers to access and utilization of PAC could have significant social, health, and economic benefits. Besides preventing severe abortion-related morbidity and deaths, access to quality PAC could also improve the high uptake of voluntary contraception among PAC clients. Limited access to quality PAC puts both young and adult women at risk of repeat unintended pregnancies and unsafe abortions [19–21], delays in receiving critical care, higher costs of care, including catastrophic expenditures, and extreme reproductive disabilities resulting from complications not treated on time. So far, most studies have focused on analyzing PAC utilization from the patient-provider perspective and not much from the health system perspective. The health systems perspective offers unique insights into the strengths and weaknesses within health facilities while providing PAC. In addition, this would also guide on specific gaps and entry points for critical interventions that address the nexus between post-abortion contraceptive uptake, unintended pregnancy, and unsafe abortion, including PAC clinical training and mentorship; service reorganization, equipment provision, and an expanded method mix offering; use of standardized PAC registers; and community engagement for awareness building and linkage to PAC. Continued gaps in the research exploring barriers to PAC inhibit evidence-based action and strategy at multiple decision-making levels to effectively address the maternal health challenges related to abortion. In this paper, we explored the barriers to providing quality post-abortion care in Burkina Faso, Kenya, and Nigeria and present robust comparative evidence on PAC services.

## Methods

### Study context

This study was part of a multi-country project to assess the quality of PAC in public health facilities in three SSA countries - Burkina Faso, Kenya, and Nigeria [22]. These three countries present unique contexts for assessing the barriers to access and utilization of post-abortion care services. Abortion is legally prohibited in the three countries, and the health systems are also largely structured similarly in all three countries, with a broad focus on primary, secondary, and tertiary health system structure [23].

### Study design and population

This paper drew data from three but linked sources—health facility assessment data, patient-exit interview data, and qualitative interviews conducted with healthcare providers and policymakers. Data were collected using a cross-sectional service provision assessment conducted among a nationally representative sample of primary, secondary, and tertiary-level health facilities in the study countries and patient-exit interviews among patients treated for post-abortion complications across all the selected facilities. Across the three countries, primary health facilities are the lowest levels of care and first point of contact for most community members needing medical attention and include community health units, dispensaries, and clinics. Secondary facilities are district/sub-regional level facilities that receive referrals from primary facilities. They undertake curative and rehabilitative care and address a limited extent of preventive/promotive care. Tertiary facilities are mainly national referral and teaching facilities. In this paper, both secondary and tertiary facilities were categorized as referral facilities. Our sample frame for health facilities included all that could conduct normal deliveries. For the health facility assessment survey, across most major facilities, managers or an appropriate designate (either an obstetrician and gynecologist, midwife, or any other health personnel) were recruited and enlisted to respond to the health facility post-abortion care service survey. However, within lower-level facilities, a nurse, a midwife, or another health worker in a position to provide information on abortion care offered in that facility was interviewed.

Patient-exit interviews targeted women and girls treated for abortion-related complications across the same sample of health facilities selected for the health facility assessment survey. Women and girls presenting at these facilities were approached at discharge and recruited to be interviewed about their experiences during care. Data was collected in each facility over 30 days between November 2018 and February 2019.

For the qualitative interviews, we targeted a sample of health providers delivering PAC across the various facility levels and regions. The respondents differed from those interviewed in the facility service assessments. Similarly, several decision-makers and stakeholders were identified based on their knowledge of the PAC landscape in the study countries and interviewed.

### Sampling and recruitment

In each country, sampling was stratified based on the highest sub-national administrative units (counties in Kenya, states in Nigeria, and regions in Burkina Faso) and by level of facilities. We selected multiple sites to account for greater diversity in demographics, cultural backgrounds, geographical locations, religion, and management of healthcare, as research findings can have policy implications that vary from one region to another. An updated master list of health facilities in the different sub-national units was obtained from the respective ministries of health. After that, we applied a two-stage sampling procedure to select the health facilities. Initially, a random sample of six regions, states, or counties was drawn in each country, excluding the unit hosting the national capital–i.e., Centre in Burkina, Nairobi in Kenya, and Abuja–Federal Capital Territory (FCT) in Nigeria. After that, the capital regions were added to the regions purposely to make seven regional units in each country. The selected administrative units were Burkina Faso: seven regions of the 13: Boucle du Mouhoun, Cascades, Centre, Centre-Ouest, Centre-sud, Haut-Bassins, and Nord. In Kenya: the seven counties from the 47 included Garissa, Kajiado, Kiambu, Laikipia, Mandera, Migori, and Nairobi. Seven states were selected from the 36 in Nigeria, including Anambra, Bauchi, Cross River, Edo, Kogi, Kano, and Federal Capital Territory (Abuja).

In the second stage, a requisite sample of facilities in each country was determined using a formula for known populations and known proportion estimates:

$$n={\left(\frac{z}{\Delta }\right)}^{2}p\left(1-p\right)$$

In this case, the known estimate *p*, was the proportion of facilities capable of providing counseling and information on techniques for preventing contraceptive method failure. This is the lowest measure of quality of care from a recent survey in Kenya (16). Unfortunately, we found no recent similar studies in Nigeria and Burkina Faso. We, therefore, used Kenya’s estimates to proxy for the other countries, given the overall similarities in health services. Once an appropriate sample was determined, this was allocated to each of the seven administrative units depending on the population of eligible facilities in a specific region. For a facility to be eligible for this study, it had to be a public health facility within a functional care level—obstetric care capable of providing and operational during the study period. In this case, specialized facilities such as mental and spinal hospitals and military and prison hospitals known not to offer services to the general public were excluded. We focused on public health facilities because government investments in health services primarily go to these facilities. During the survey implementation, some facilities were dropped due to insecurity and inaccessibility and replaced with similar facilities within the same locality. Also, sampled facilities that declined to participate in the study were replaced with similar facilities from the sampling frame. After accounting for a response rate of approximately 93%, requisite samples of facilities were determined as 414 in Burkina Faso, 259 in Kenya, and 223 in Nigeria.

For the patient-exit interviews—trained research assistants stationed at each health facility recruited all women who presented for post-abortion care at the health facilities. Once they consented, they were interviewed in the study. A total of 2174 women who received PAC were interviewed in Burkina Faso, 819 in Kenya, and 1247 in Nigeria during the 30 days of health facility observations.

All respondents (health providers and decision-makers) in the qualitative interviews were selected purposely because they know PAC service delivery in health facilities and the countries’ PAC policy, strategy, and service landscape.

### Data collection and analysis

Trained research assistants (primarily professional clinicians—nurses and midwives) visited each sampled facility. They administered the health facility assessment questionnaires, and other research assistants (non-clinicians) conducted the patient-exit questionnaire interviews. The health facility assessment questionnaire was a signal function tool adapted from Campbell et al. and Healy et al. templates. The adaptations resulted from extensive discussions by researchers and experienced obstetricians and gynecologists in each country [24,25]. Respondents at large referral hospitals were the heads of the obstetrics and gynecology department or a key obstetrician/gynecologist working in the facility. However, a nurse or midwife knowledgeable on PAC services was interviewed at lower-level facilities. Data was collected using tablets and hosted on the SurveyCTO application platform. For the patient interviews, the tool was adopted from the frameworks developed to advance person-centered care for reproductive health equity by Sudhinaraset and colleagues [26]. Research assistants assigned to each health facility conducted these interviews. All women treated for abortion-related complications in the selected facilities and agreed to participate were included in the study until the 30-day observation period was achieved. The use of non-providers to capture the patient-exit data was to avoid bias and patients feeling intimidated while being interviewed by providers. All PAC clients who consented to participate in the study, were recruited as participants.

To explore the perspectives of healthcare providers and patients on the status of PAC services in health facilities- 50 in-depth interviews (IDIs) were conducted with healthcare providers in Burkina Faso, 54 in Kenya, and 65 in Nigeria. Selected health providers reflected variations in facility levels, professional cadre, ability, or inability to provide PAC across various locations. All interviews were captured using a digital recorder.

Data analysis was conducted using Stata version 16.1. Much of the analysis of how post-abortion care is assessed and quantified across the three countries has been documented in a previously published paper [22]. For this specific paper, we summarized the proportions of facilities incapable of delivering particular service indicators across the countries and facility levels. Patient experiences on specific domains such as stigma and discrimination, privacy, and confidentiality were described using proportions and presented in figures and tables. Qualitative data were analyzed using a grounded theory approach.

### Ethical considerations

The study protocol was reviewed and approved by the AMREF Ethics and Scientific Research Committee (ESRC) **(protocol ID: AMREF-ESCR P429/2018**) and the University of Nairobi/Kenyatta National Hospital Ethics and Research Committee **(protocol ID: KNH-ERC/A/384)** in Kenya. Permits to conduct the study were also obtained from the Kenyan National Commission for Science, Technology, and Innovation (NACOSTI) and each participating health facility. The study protocol was also approved by the National Health Research Ethics Committee of Nigeria (NHREC) **(protocol ID: NHREC/01/012007-20/08/2018)** and in Burkina Faso by the Health Research Ethics Committee (CERS) **(Protocol ID: 2018-10-124**), as well as authorization letters from the ministries of Health in all the countries. Signed consent was individually obtained from each participant participating in the study, and the interviews were done privately.

## Results

Nine hundred health facilities were sampled across the three countries, and 894 responded to the PAC service assessment tool, with 414 in Burkina Faso, 253 in Kenya, and 227 in Nigeria. Most Burkina Faso and Kenya facilities were primary-level facilities; in Nigeria, most were referral-level facilities. Additional details on the health facilities that participated are found in Table 1.

**Table 1:** Health facilities by countries.

	Primary-level facilities n (%)	Referral-level facilities n (%)	Total (facility response rate) n (%)
Burkina Faso	354 (85.5)	60 (14.5)	414 (100)
Kenya	211 (83.4)	42 (16.6)	253 (97.6)
Nigeria	92 (40.5)	135 (59.5)	227 (100)

Typical services offered by the various levels of facilities.

**Primary-level facilities** provide promotive and preventive care and various curative services, including prenatal, delivery, and antenatal services. **Referral facilities**: undertake curative and rehabilitative activities, to a limited extent preventive/promotive care, and are a referral point for primary facilities.

### Training of health care providers on PAC

**Fig 1:**
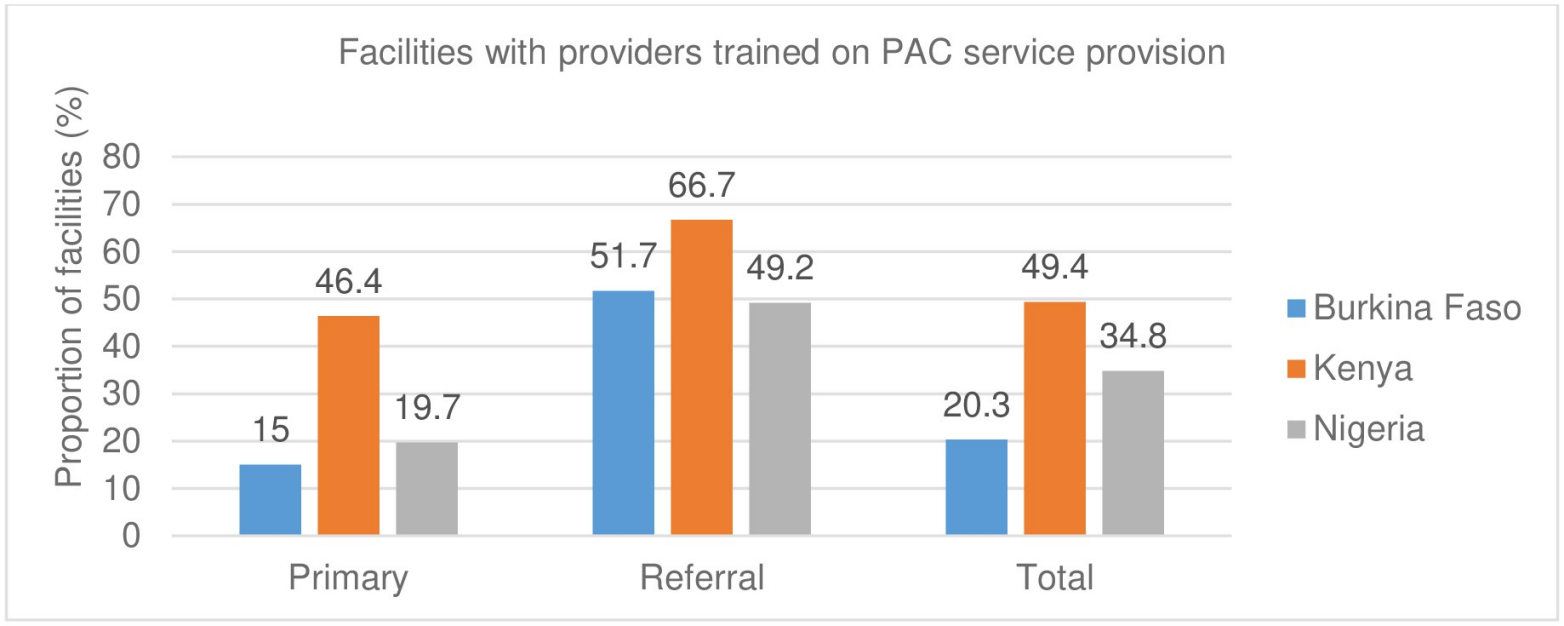
Facilities with providers trained on comprehensive PAC provision.

The proportion of staff trained on PAC was generally low across the three countries. Less than half of health facilities had staff trained on comprehensive PAC across (Kenya 49.4%, Nigeria 35%, and Burkina Faso 20.3%). Further, fewer primary-level facilities in Burkina Faso (15%), Kenya (46%), and Nigeria (20%) had staff trained on PAC compared to referral-level facilities (Fig 1).

In-depth interviews with health providers confirmed deficiencies in PAC training to health providers. Providers indicated relying on pre-service training (received while in school) to handle "non-complicated" cases. According to the providers, this training was insufficient to enable them to offer comprehensive quality PAC to all patients needing such services.

*Honestly*, *the major problem we have here is the [lack of] training*. *Look*, *in an entire maternity ward of a CMA [Centre Medical avec Antenne chirurgicale]*, *there is no one trained in PAC*, *except the basic training we got at school*. *For instance*, *the use of misoprostol*, *I was never trained*. *As a result*, *we are forced to train ourselves by getting information from the Internet or from other colleagues*, *just to be able to help the patients*

(Midwife, secondary level facility, Burkina Faso)

Providers lamented that they are forced to learn on the job and, at times, draw from internet sources. They also rely on their contemporaries, their seniors at the facility, or those trained on PAC provision. Even so, most providers admitted that they always refer most patients they consider complicated to higher-level facilities.

…, *for a while now we have not had official training*, *and it will be nice to have it*, *most of the training is when a younger staff works with the experienced ones*, *there she learns*, *but you know if you are talking about the training for PAC we have not done any training on* that

(Nurse, tertiary facility, Nigeria)

Health providers with PAC training were rarely available throughout the health facilities. Similarly, the absence of comprehensive PAC training meant that even staff trained still needed to gain adequate skills to treat and manage a range of post-abortion complications. In addition, even when training is offered, the training sessions are infrequent, and there are high staff turnovers and transfers across facilities, creating a critical and persistent skill gap.

*The doctor is not always around*, *when such cases come*, *we just refer because I have not been trained*. *We are three here and none of us was trained*. *We refer because it is a critical situation*. *Sometimes the woman can bleed*, *so I don’t want to start something I can’t finish*

(Nurse, secondary facility, Nigeria)

Some gaps in the training of PAC providers could be attributed to inconsistent policies and clinical guidelines around PAC provision, especially in Kenya, where the guidelines provide for routine training of health providers but need to provide detailed information on the implementation framework for such training.

*You know initially we used to have the guidelines on the post-abortion care but somewhere from twenty twelve (2012)*, *there was a circular that came from the DMS [Directorate of Medical Services] that cancelled all those things such that we cannot even train the health care providers*

(Senior MoH official, Kenya)

#### PAC equipment

Our assessment revealed that most facilities lack essential equipment and supplies to deliver PAC (Table 2). For instance, 16.6% of health facilities had a functional operating theater or an MVA room in Kenya and Burkina Faso (20.3%); in Nigeria, it was slightly more than half (50.7%). Power availability, including a backup generator in case of a power blackout, was only reported in 22.5% and 28.5% of Burkina Faso and Kenya health facilities, respectively. In contrast, in Nigeria, it was 62.6% of the facilities. Most (88.4%) facilities in Burkina Faso had a functional landline or mobile phone for calls in case of emergency; in Kenya, it was 56.9% facilities and 16.6% in Nigeria. Within the PAC treatment room, 67.6% and 50.2% of Kenya and Nigeria facilities had specific suction tubes, while in Burkina Faso, only 15.2%. As for vacuum aspirator kits or syringes, less than half the facilities in Burkina Faso (33.1%), Kenya (36.4%), and Nigeria (47.1%) had these on hand. Only 24.4% of facilities in Burkina Faso, 53.8% of facilities in Kenya, and 49.8% of facilities in Nigeria had a manual vacuum aspiration (MVA) kit. More than half of the facilities (54.6%) in Burkina Faso, 67.2% in Kenya, and 67.8% in Nigeria had an examination light or a light source for use during PAC.

**Table 2:** PAC equipment and supplies.

Equipment	Burkina Faso n (%)	Kenya n (%)	Nigeria n (%)
**The following instruments, equipment, and supplies are needed for PAC, n (%)**			
Vorsellum/Tenaculum	88 (21.3)	199 (78.7)	103 (45.4)
Sponge (ring) forceps	49 (11.8)	218 (86.2)	134 (59.0)
Vaginal speculums	329 (79.5)	242 (95.7)	144 (63.4)
Needles and syringes	375 (90.6)	245 (96.8)	206 (90.8)
Manual vacuum aspiration pack	101 (24.4)	136 (53.8)	113 (49.8)
Examination light/ Light source or flashlight	226 (54.6)	170 (67.2)	154 (67.8)
Disposable latex gloves (surgical)	383 (92.5)	246 (97.2)	197 (86.8)
**The health facility or the treatment room should have the following furniture and equipment in working order, n (%)**			
Functional operating theatre/MVA room	84 (20.3)	42 (16.6)	115 (50.7)
Examination/Procedure bed	370 (89.4)	249 (98.4)	186 (81.9)
Functional Landline/Mobile phone in the facility	366 (88.4)	144 (56.9)	35 (15.4)
Electricity (e.g., electricity grid, generator, solar)	367 (88.7)	226 (89.3)	155 (68.3)
Backup generator (in case of power outage)	93 (22.5)	72 (28.5)	142 (62.6)
A toilet (latrine) on premises (functioning)	389 (94.0)	239 (94.5)	178 (78.4)
Stethoscope	362 (87.4)	249 (98.4)	211 (93.0)
Suction catheter, 10, 12	63 (15.2)	171 (67.6)	114 (50.2)
Suction aspirator operated by foot/electronically	86 (20.8)	172 (68.0)	103 (45.4)
Vacuum aspirator kit/syringes	137 (33.1)	92 (36.4)	107 (47.1)
Private room for examining/treating/counseling	365 (88.2)	250 (98.8)	199 (87.7)
Ultrasound scanner	47 (11.4)	26 (10.3)	83 (36.6)

**Fig 2:**
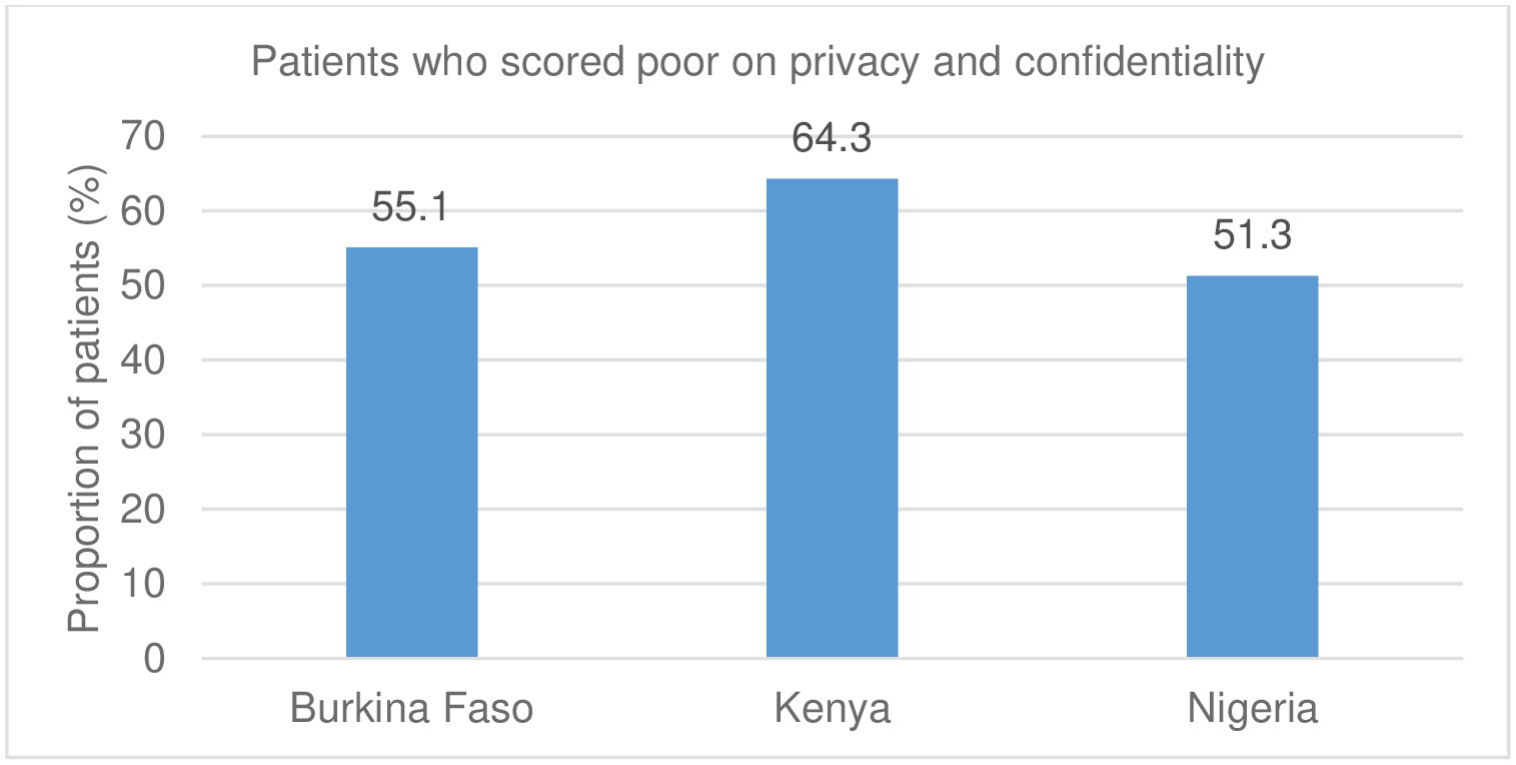
Patients with a poor score on privacy and confidentiality.

The absence of a specific MVA room in most facilities meant that MVA procedures are conducted in non-designated rooms and spaces that may lack the necessary privacy and tools for this service. This finding was associated with poor scores recorded during the patient-exit interviews on privacy and confidentiality. More than half of the patients in Burkina Faso and Nigeria and over 64% in Kenya felt they were not guaranteed privacy or confidentiality during treatment (Fig 2).

Health providers noted that the lack of PAC equipment and supplies crucial for the management of "incomplete abortions" or "hemorrhagic abortions" often hinders quality service delivery. Even in situations where PAC is free, for instance, in Burkina Faso, shortages and frequent stock-outs of critical medical supplies often compel patients to purchase the required drugs to treat their complications.

…*Often there are shortages too*. *(*…*) In any case*, *we prescribe*, *the patients go outside to see if they can pay*

(Midwife, Secondary level facility, Burkina Faso)

As emphasized by a provider in Kenya, the lack of equipment or commodities was more prevalent in primary-level facilities, where providers lacked MVA kits or rooms, ultra-sonographers, observation rooms, blood banks, and other equipment. In some facilities, MVA kits were never supplied, while in others, restocking was delayed, leaving such facilities in "*a sorry state*.*"*

*I don’t know when it was supplied because there are those cannulas [tubing inserted into a vein or body cavity to aid in administering medication or fluids*, *drain fluid*, *or insert surgical instruments]*, *they are color coated*, *the smallest being the yellow one*. *It is not here; so it means you have to use the larger ones*. *So*, *sometimes I have challenges*. *Actually*, *I’ve never used it from the time I reported here"*

(Clinician, primary healthcare facility, Kenya)

**Fig 3:**
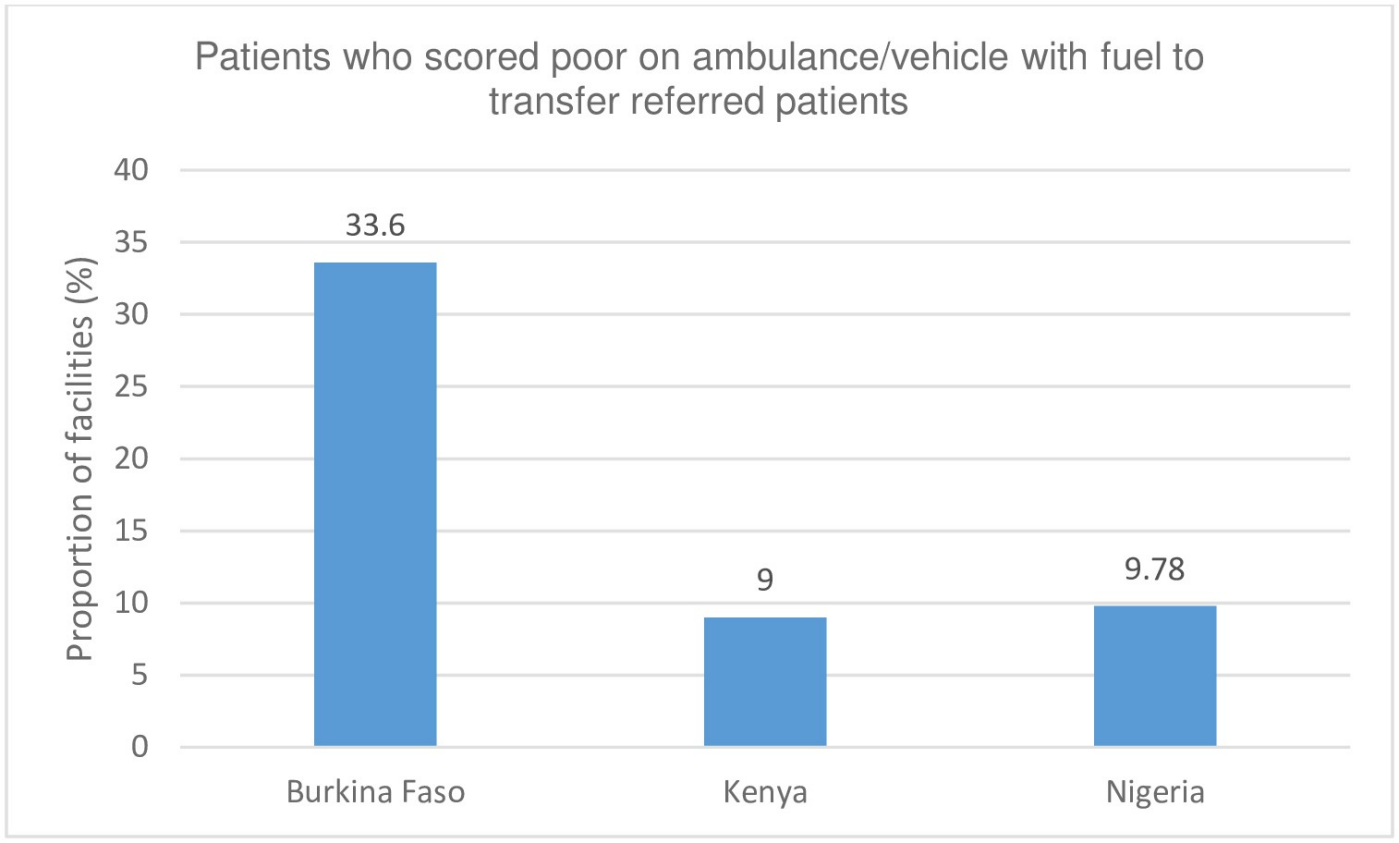
Facilities with ambulance/vehicle with fuel to transfer referred patients.

The referral capacity within the primary-level facilities was deplorable across the three countries. More specifically, the direst situations were observed in Kenya and Nigeria, where only 9% and 9.78% of facilities had an ambulance/vehicle with fuel to transfer referred patients, respectively (Fig 3).

Qualitative interviews revealed that most primary-level facilities need more training and equipment to deliver PAC. Therefore, most of them often refer PAC patients to other higher-level facilities. For effective referral, providers emphasized that the presence of an ambulance is critical for transporting patients in need of medical attention from higher-level facilities. However, they noted that most primary-level facilities have none and rely on referral-level facilities for ambulances. As such, ambulances are only sometimes available or may take a long time to come to facilities located in distant areas. Moreover, facilities with ambulances reported recurrent breakdowns, as explained by one participant in Burkina Faso:

*We only have one ambulance*, *but often*, *the ambulance is in the garage*. *Yesterday even until I left the facility*, *the ambulance was still in the garage*, *yet we are a referral facility*

(Maieutician, referral level facility, Burkina Faso)

As shown in Table 2, most health facilities lack an ultrasound machine. As such, PAC patients are often given an ultrasound scan prescription/referral to another facility for the service. This implies increased costs to clients in scanning and transportation costs and greater delays in getting appropriate care.

*I came back with papers here to show the woman and they said that the pregnancy is spoiled*, *they told me they have the medicine [misoprostol] that costs 5000f*. *When I left home*, *I had 10000f and the echo took 8000f*, *I didn’t have someone there to supplement my money to pay for the medicine which is 5000f*. *After three days we went back to pay for the medicine*, *I came here to show the woman the medicine I had paid for and she told me to go home that the blood will come out*

(29, married, trader woman, rural, Burkina Faso)

Additionally, most patients recounted that they were forced to have an ultrasound performed on them in the facility from which they were seeking care for them to be treated or referred. This happened even when they had done the scan elsewhere and had it with them.

*When I entered*, *I met one doctor and explained to him that my baby is not moving and they asked me for the paper [ultrasound] to prove what I told*, *I gave them the paper but they said that I should go and do the scan in their own facility to confirm it*

(29 years, separated, cleaner, urban, Nigeria)

#### Patient-provider interactions

**Fig 4:**
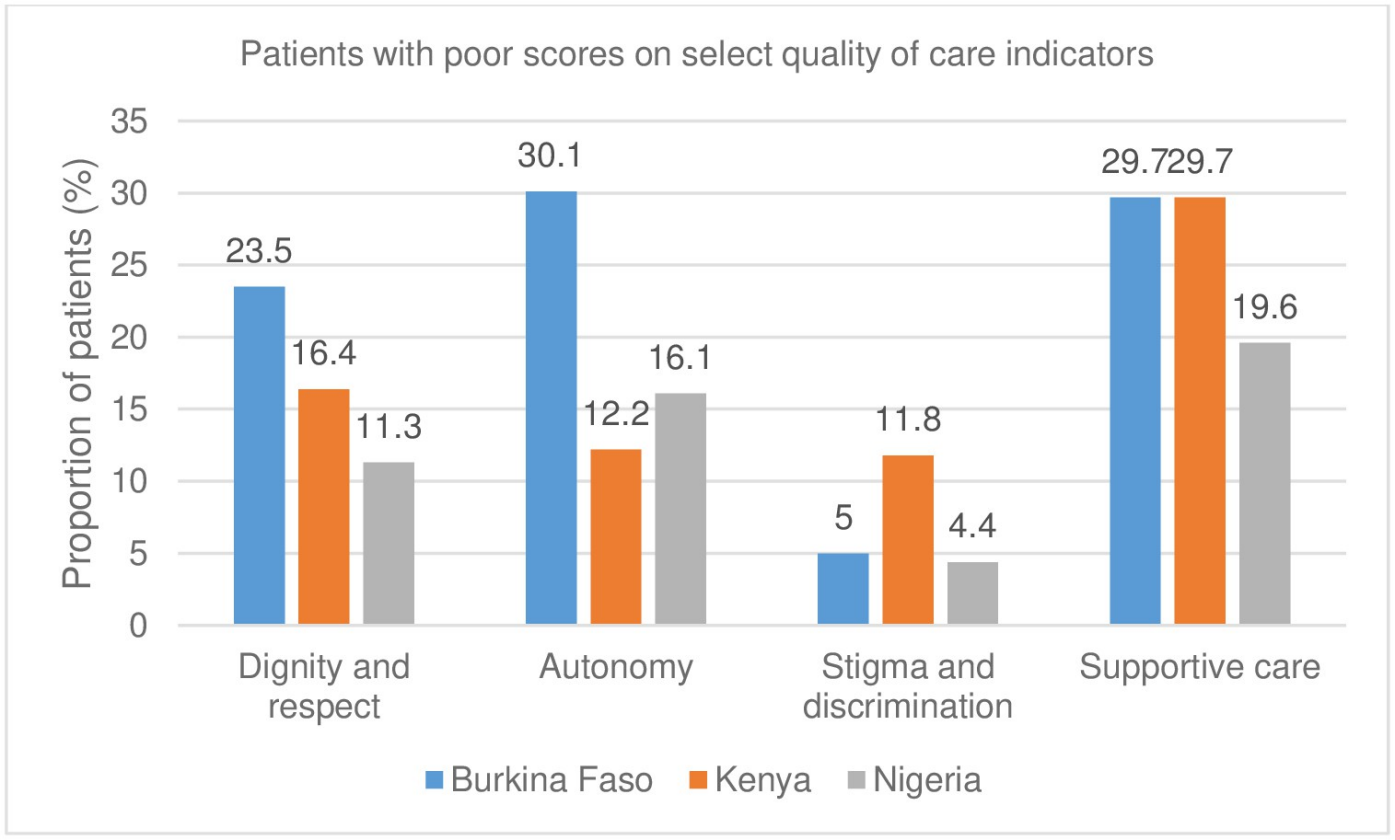
Patients with poor scores on select quality of care indicators.

Regarding patient-provider interactions, a considerable proportion reported poor scores on dignity and respect and the ability of women to receive care in a respectful and caring setting in Burkina Faso (23.5%), Kenya (16.4%), and Nigeria (21.3%). Autonomy, meant to capture patients’ involvement in the decision-making regarding their care, equally scored poorly: 30.9% in Burkina Faso, 22.8% in Kenya, and 16.1% in Nigeria. Concerning stigma and discrimination, 10.8% in Kenya scored poorly, while in Burkina Faso and Nigeria, 5% and 4.4%, respectively. Supportive care was explored by examining whether health providers provided care in a timely, compassionate, and caring manner, as well as integration of care in a way that is responsive to patient needs; 29.7% in both Burkina Faso and Kenya and 19.6% of participants in Nigeria rated this poor (Fig 4).

Most of the patients interviewed conveyed that they were less involved than they would have preferred in decisions about their care. Though they reported that they were informed about their conditions, they were not consulted on the care process, as explained by one patient in Burkina Faso:

*I*: *In relation to the treatments received*, *have you been asked for your opinion*?

*R*: *No*, *I thought they would allow me a little time for the cervix to open on its own*. *But they just came and gave me tablets to take*

(Married, employed, Burkina Faso)

In-depth interviews revealed that some patients experienced stigma and discrimination. This was experienced mainly by patients who reported hostility and impoliteness while interacting with providers. One patient whose care was delayed and experienced providers’ apathy felt she was treated in a demeaning manner because of her attributes, namely being young and suspected of having induced her abortion:

*Well since this is my first pregnancy*, *and being a young girl also*, *even if I tell them that it’s not induced*, *it’s hard for them to believe me*. *Some will say that no*, *girls nowadays*, *take products (*…*)*. *I mean*, *when I was in that room*, *there were some people who come after us*, *but they got care and left us*

(24 years old, single, Burkina Faso)

Health providers’ values and personal bias were also pointed out as a deterrent to accessing PAC services. Respondents highlighted instances where their colleagues were reluctant to offer services because of religious-related beliefs. As an alternative, these providers referred the patients to other colleagues or facilities, thus delaying access to care and increasing the risks from complications.

*Depending except one two staff who have a religious issue on*, *relating to general FP and PAC issues*, *generally the others are okay*. *It affects in one way because*, *one she will not be able to provide the PAC service so meaning it becomes a problem to the client because that client will be told wait for so and so to come so that you are able to be provided the service and remember in PAC that delay would also not be good for the patient*

(Nurse, primary healthcare facility, Kenya)

Even though supportive care was provided to most of the patients who went through spontaneous abortion, some patients reported cases of rudeness, verbal abuse, or lack of attention, especially when they were suspected to have an induced abortion. This is described by one of the participants:

*Well*, *sometimes you talk to them and they don’t even listen*.…*When I was in pain*, *I woke up*, *stood at the door*, *and talked to them*. *They looked at me*, *but nobody answered me*, *yet they saw me suffering…*. *Sometimes they are just there*, *just chatting*, *and not caring about people*. *(*… .*) Since it’s my first time to get pregnant*, *as I’m a young girl too*, *even if I tell them that I did not induce it*, *it’s hard for them to believe me*.

(24 years old, single, student, urban, Burkina Faso)

## Discussion

This study aimed to describe the barriers to the provision of post-abortion care services across three SSA countries—namely Burkina Faso, Kenya, and Nigeria. Our analysis showed remarkable gaps and challenges within health facilities impeding the delivery and access of this essential reproductive health service. Overall, there were rampant reports of absence or an inadequate number of trained providers on PAC, absence of PAC infrastructure, equipment, and supplies, as well as poor patient-provider interactions. Most primary-level facilities could not offer quality PAC services even though they are where patients seek care first [27].

Very few primary and secondary health facilities had providers trained in delivering PAC services across the three countries. The limited number of providers trained within health facilities means they are only available at a particular time yet PAC is an emergency service. While training is vital in providing services [28], its facilitation or lack thereof is greatly determined by guidelines and policies primarily informed by the existing legal framework. Lack of skills implies that providers offer ’skeleton services’ drawn from their in-school training, which is often basic. Importantly, it is critical to note that the lack of implementation (where they exist) creates uncertainty about what services providers can offer based on legality. This is also an avenue through which stigmatization is perpetrated, as most providers can hide behind that as their reason for declining to offer services or substandard provision. Consequently, this affects the completeness and compromises the quality of care given. This means patients do not receive a complete care package since the provision of PAC has many components, including evacuation of retained products of conception and psychological and contraceptive counseling. [29].

Prior studies have highlighted the importance of adequate referral systems in filling the gap of facilities/providers’ inability to offer services, hence bringing services closer [30], and that for healthcare service delivery to be effective, programs have to be designed and implemented in a way that takes into account the referral system [31]. Since the capacity to deliver various PAC services was very low, most facilities referred patients immediately, even without offering emergency treatment or counseling. However, this study has shown the limited effectiveness of PAC referral chains in the three countries. The capacity to refer patients in a critical state–reflected by the availability of an ambulance or a vehicle at the facility (with fuel) was deficient. Therefore, most patients referred have to facilitate their referral. In this case, referral means additional cost of care and may lead to delays with poor health outcomes for the woman. In addition, most patients do not complete their referrals due to the stigma associated with abortion. They would prefer to have all services in one place, not different locations and providers, to minimize their exposure. Referral facilities are also always overcrowded, and as a result, one is likely to result in poor service delivery since the huge patient numbers strain providers. In these instances, PAC patients are compelled to use private transport, regardless of whether they can afford it or not and their conditions allow it.

Poor experiences by patients were mainly reported in referral-level facilities where the high volume of patients (partly due to referrals) does not allow for sufficient time for providers to interact effectively with the patients [32]. Patients complained of being treated disrespectfully, insulted, and verbally abused by providers; they felt exempted from decision-making about their care and experienced discrimination. Previous studies have shown that patients feel more comfortable if their privacy, including needs, attitude, and concerns, are respected and guaranteed during examinations, procedures, and counseling [33,34]. Our findings reveal that patients were apprehensive about their privacy and confidentiality, which can be linked to the lack of an exclusive MVA room. Most facilities had improvised and were using other rooms for the procedure. Patients are, therefore treated in shared rooms, compromising their privacy, confidentiality of their condition and procedure, as well as their psychological wellbeing, especially for those being treated for miscarriage (pregnancy loss). Even though patients’ involvement in their care (autonomy) is a vital aspect of quality of care, a good number of the patients reported that they were not involved in the decision-making process regarding their care, such as the choice of the protocol for evacuating the conception product. Some patients said that providers, especially at the referral level, paid little attention to them during admission and to their cries when they needed support or help.

### Limitations

The exclusion of private health facilities is a limitation. Indeed, PAC services are also available in private facilities across the three countries; our findings did not cover women seeking PAC services from private facilities. It is essential to note the potential influence of courtesy bias on participants’ responses as they might not have been at liberty to give negative feedback about the care they received. Also, the lack of data on patient outcomes implies we could not tie these barriers to PAC provision to ultimate outcomes.

## Conclusions

Despite governments’ commitments to ensure PAC services’ availability, there are numerous impediments to the provision of quality care. While PAC is a critical emergency service, results from this study point to severe gaps and weaknesses in the delivery of PAC in Kenya, Nigeria, and Burkina Faso. These gaps can be attributed to the lack of clear policy guidelines to an inadequate number of providers due to the low numbers trained on PAC, high staff turnover, lack of equipment, inadequate supply of commodities, and inadequate spaces for treatment that guarantee privacy. To achieve improved maternal mortality targets, governments need increased investments to strengthen the capacity of health facilities to deliver quality PAC services, including training of health providers, supplies and commodities, and referral to higher-level facilities.
